# Extending the concept of moral distress to parents of infants hospitalized in the NICU: a qualitative study in Greece

**DOI:** 10.1186/s40359-024-01793-8

**Published:** 2024-05-24

**Authors:** Polychronis Voultsos, Maria Arabatzi, Maria Deligianni, Alexandra K. Tsaroucha

**Affiliations:** 1 Laboratory of Forensic Medicine & Toxicology (Medical law and Ethics), School of Medicine, Faculty of Health Sciences, Aristotle University of Thessaloniki, University Campus, Thessaloniki, GR 54124 Greece; 2 Postgraduate Program on Bioethics, Laboratory of Bioethics, Laboratory of Experimental Surgery and Surgical Research, School of Medicine, Democritus University of Thrace, Dragana, Alexandroupolis, GR 68100 Greece

**Keywords:** Neonatal intensive care unit (NICU), Parents, Uncertainty, (Shared) Decision-making, Ethical dilemma, Moral distress, Constraint distress, Uncertainty distress

## Abstract

**Background:**

The hospitalization of infants in the neonatal intensive care unit (NICU) is an ethically challenging situation. A limited number of studies have extended the concept of moral distress to parents of infants hospitalized in the NICU. This topic requires further investigation.

**Methods:**

The present prospective qualitative study was conducted from February 2023 to May 2023. Data were collected through semistructured in-depth interviews, which were conducted in-person with fifteen parents of infants who were hospitalized in the NICU at the time of the interviews. Purposive sampling was used. The data were classified and analyzed using thematic analysis.

**Results:**

Three themes emerged from the data analysis performed for this empirical study. One intrapersonal dimension featuring two aspects (one dynamic and one static) and another interpersonal dimension focusing on parental moral distress emerged from the data analysis. Furthermore, seven subthemes emerged across these themes: (1) self-directed negative feelings were experienced by parents due to their inability to fulfill their caregiving/parental roles; (2) intense internal conflict was experienced by parents in response to a moral dilemma that was difficult, which was perceived as irresolvable; (3) objectively unjustified, self-directed negative feelings of guilt or failure were experienced by parents; (4) parents experienced moral distress due to the poor image of the ill infants; (5) inadequate information may predispose parents to experience moral distress (6) neonatologists’ caring behaviors were unduly perceived by parents as paternalistic behaviors; (7) reasonable or justified institutional rules were unduly perceived by parents as constraint.

**Conclusions:**

In general, the results of this study support the integrated definition of parental moral distress proposed by Mooney-Doyle and Ulrich. Furthermore, the present study introduces new information. The study distinguishes between the dynamic and static aspects of the intrapersonal dimension of the phenomenon of parental moral distress. Moreover, participants experienced moral distress because they unduly perceived certain situations as causing moral distress. In addition, inadequate information may predispose parents to experience moral distress. The findings of this study may contribute promote family-centered care in the NICU context.

## Introduction

The hospitalization of infants in the high-tech and highly specialized neonatal intensive care unit (NICU) environment is an inherently traumatic and stressful procedure for parents. Mental distress is prevalent among parents of infants in the NICU [1–3]. This situation may cause them to experience a “cluster” of symptoms related to depression, anxiety, trauma, posttraumatic stress or even guilt [4–7]. Mooney-Doyle and Ulrich [5] provided the best characterization of this situation when they noted that “parental physical and emotional distress is exhibited in many ways, including fatigue, disrupted sleep, caregiving demands, worry, and employment demands”. In NICUs, complex dilemmas commonly and regularly emerge, and ‘decisions regarding end-of-life care, periviable resuscitation, and medical futility’ must be made [8]. Furthermore, neonatal intensive care involves ethically challenging situations that give rise to complex ethical dilemmas in a context involving a high degree of uncertainty [8, 9], especially with regard to extremely premature infants (EPIs) who are born at the limit of viability (i.e., weeks 22–25 of gestation). As parents become increasingly active in the medical care of their infants, they may struggle to make value-laden judgments in the traumatic NICU environment [8, 10, 11].

Ethical dilemmas, uncertainty, and moral distress are closely related albeit distinct concepts [8, 12]. Uncertainty may lead to moral distress if core values and moral integrity are compromised [8, 12]. It has been argued that moral dilemmas occur when principles or values conflict, and the decision-maker must “violate at least one important moral concern” [9] by choosing between “mutually inconsistent courses of action” [13], which are ‘equally unsatisfactory alternatives’ [14]. “Moral uncertainty arises when one is not sure whether there is an ethical dilemma, or when one assumes that there is an ethical dilemma but is not sure what principles or values apply in the ethical conflict” [15]. Importantly, in neonatology, various moral dilemmas emerge in contexts involving extreme uncertainty, such as (1) end-of-life care and palliative care, (2) medical utility and futility, (3) per viability, and (4) conflict and disagreement [8]. Moral distress is highly prevalent in the NICU context [8]. This phenomenon is conceptually distinct in the clinical context and was first defined by Jameton as “the psychological distress of being in a situation in which one is constrained from acting on what one knows to be right” [16, 17]. This situation inherently challenges people’s ethical principles and values [18]. Health professionals have “little power to act differently or change the situation” [18] or have “no choice but to act this way” [19]. This “constraint distress” is the original/narrow definition of moral distress. Constraints are either external constraints (i.e., institutional rules, hierarchy of financial factors, fear or other circumstances that lie beyond the health care professional’s control) [20] or internal constraints [5, 12]. According to Deschenes et al., “External constraints are widely described as obstacles outside of the individual, whether institutional, systemic or situational, while internal constraints are located within the individuals themselves and are described as personal limitations, failings or weakness of will” [21]. Health care professionals can be constrained from taking the correct action or compelled to do what he or she ‘believes is the wrong thing’, and their attempts to do the right thing may even fail [20]. They feel as if they are acting against their deeply held professional or personal values [20].The negative psychological impacts include anguish, suffering, ‘psychological disequilibrium and negative feeling states’, and self-directed emotions such as a ‘feeling of powerlessness’ that ‘impels him or her to accept imposed individualities, have his or her resistances reduced…and obstructs the process of moral deliberation, compromises advocacy and moral sensitivity’ [20]. Health professionals may be constrained not only from acting on what they know ‘to be right’ but also influenced by their sense of moral responsibility or their desire to preserve all ‘interests and values at stake’ [20].

Nevertheless, the definition of moral distress has been revised (reconceptualized) and expanded in recent years. In this regard, moral distress has come to be understood as the individual’s specific psychological response to morally challenging/troubling/undesirable situations such as those involving moral constraint, moral conflicts/dilemmas or uncertainty [12, 22], i.e., situations which compromise the individual’s values and moral integrity [5, 22]. For many years, a variety of definitions of moral distress have been offered, which have largely focused on “constraint” and/or “uncertainty”, thereby highlighting various aspects of this phenomenon [20].

Definitions tend to blur the distinction between moral distress and uncertainty (or even dilemma). This conceptualization undermines the concept of moral distress as a conceptually distinct concept and leads to methodological problems [21] with regard to research on this phenomenon and the development of prevention strategies. Certain morally relevant lines of distinction should be observed. We should avoid neglecting these distinctions.

While moral distress was originally conceptualized as a phenomenon involving health care professionals, initially including nurses and then encompassing all health care professionals [12, 23]; it is now considered to be a phenomenon that is not restricted to health care professionals but rather transcends them and affects families [5, 8, 23, 24]. A (limited) number of studies have mentioned moral distress among parents of infants hospitalized in the NICU [5, 8, 24]. Mills and Cortezzo argued for a broad definition of parental moral distress that encompasses the parental distress that may arise from morally troubling or undesirable situations in addition to those associated with constraint, which challenge values and compromise their moral integrity [5, 8]. Foe et al. identified parental moral distress as constraint distress when parents are involved in making value-laden decisions in the NICU [24]. These authors defined parents’ involvement in situations featuring very high levels of uncertainty that cause them to experience intense internal conflict as ‘moral schism’ [24].

As early as 2006, Campbell, Ulrich, and Grady provided a broad definition of moral distress that is applicable not only to health care professionals but also to patients’ families. As these authors stated, “Moral distress = one or more negative self-directed emotions or attitudes that arise in response to one’s perceived morally undesirable involvement in a situation that one perceives to be morally undesirable” [25]. Many years later, Mooney-Doyle and Ulrich used this definition to expand the concept of moral distress to encompass parents of ill children and claimed that “parent moral distress may be one or more negative self-directed emotions or attitudes that arise in response to a situation in which important parental roles are perceived to be threatened or their ability to enact important roles is stymied” [5].

The phenomenon of parental moral distress is conceptualized in terms of the mental distress experienced by parents who feel that they are a part of a morally troubling situation in which they feel helpless and powerless to do anything to make that situation better. This feeling must be objectively justified in the given circumstances. Similarly, Wilson et al. provided the following definition: “Moral distress refers to the emotional experience of feeling involuntarily complicit in an unethical act but have little power to act differently or change the situation” [18]. The phenomenon of so-called “parental moral distress” entails constraints of any nature whatsoever that stand in the way of parents’ attempts to rectify a morally troubling situation (in which they are embedded). Parents’ feelings of powerlessness compromise their own moral integrity or values, thereby provoking intense self-directed negative emotions. Such constraints may be internal or external (i.e., institutional or financial) and may arise within all parental relationships parents (i.e., their relationships with their ill infant, health care professionals, family members or even themselves). Simple concerns based on uncertainty do not constitute moral distress, although they are related to and overlap with moral distress.

Health professionals’ moral distress and parents’ moral distress are separate phenomena, albeit closely related and overlapping. While health professionals’ moral distress is a well-studied phenomenon, parental moral distress remains an unclear, complex and underexplored phenomenon. Many definitions of health care professional moral distress have been proposed; these definitions overlap and focus on the core concepts of “constraint distress” and/or “uncertainty distress”. However, none of those definitions seems to be sufficiently broad to capture the unique and loosely defined phenomenon of “parent moral distress” effectively. The integrated definition provided by Mooney-Doyle and Ulrich is the only definition that is specific to the phenomenon of interest and sufficiently broad to capture it. However, the components of the construct of “parent moral distress” must be defined in more concrete and precise terms. This topic requires further investigation, which must be not only theoretical but also empirical. Importantly, little empirical research on moral distress has been conducted beyond the specific group of health professionals. In the present research, we did not conduct an exploratory phenomenological study that sought to develop a concept of moral distress based on parental experiences. Rather, aimed to explore the experiences of parents of neonates who were admitted to the NICU regarding moral distress as defined by Mooney-Doyle and Ulrich. We designed a set of research questions and interview questions and conducted a thematic analysis based on the definition provided by Mooney-Doyle and Ulrich. Furthermore, as empirical research on how parents with infants in the NICU navigate ethically questionable and undesirable situations in Greece is poor, the present study aimed to offer further empirical evidence on this topic with the goal of addressing this research gap.

## Methodological aspects

### Objective

The present study involved prospective, qualitative, in depth, semistructured interviews with fifteen parents (mostly mothers) with an infant hospitalized in the NICU at the time of the interviews.

### Research question

This research sought to answer the following question, which highlights the focus of this study:

What are the negative self-directed emotions or attitudes of parents that arise due to the impact of their child’s illness on their relationships with themselves and the individuals surrounding them?

### Study design

The present research involved a qualitative empirical study based on in-depth interviews conducted with parents with an infant hospitalized in the NICU.

### Inclusion criteria

The sample of this study included parents who had an infant hospitalized in the NICU at the time of the interviews.

### Exclusion criteria

Parents who (a) said they had never faced an ethically challenging/questionable/undesirable situation regarding their hospitalized infant, (b) were not able to discuss the topic of interest at the time of the interview or (c) lacked fluency in the Greek language were excluded from the sample. Furthermore, to ensure the inclusion of participants who were involved in morally troubling situations that were similar (to a greater or lesser degree), (d) parents of full-term infants and infants with congenital anomalies were excluded.

### Participant selection and setting

A purposive sampling method was used to recruit possible participants. This study was conducted at two NICUs associated with “Hippokrateion” Hospital of Thessaloniki After receiving approval from the Scientific Committee of the hospital.

The two NICUs of the “Hippokrateion” Hospital of Thessaloniki are two of the few neonatology intensive care reference hospitals to be located in northern Greece. The diversity of the sample (which reflects the validity of the study) was verified, given that both NICUs associated with the “Hippokrateion” Hospital of Thessaloniki might feature hospitalized infants from different parts of northern Greece. Participants were contacted face-to-face and provided with information; they were then asked to confirm their participation and to determine a suitable time and place for the interviews. The sample consisted of 15 participants, including fourteen mothers and one father. The invitation to participate in the study was distributed to both parents; most fathers refused to participate in the interviews and referred us to their spouses or partners on various grounds, such as “my wife could describe things better” or “I have no time”.

### Data collection and analysis

This qualitative study was conducted from February 2023 to May 2023. The interviews lasted an average of 47 min and were conducted at interviewees’ preferred times in quiet and neutral places of their choice; only the interviewer (MA) and the participant were present. Field notes were taken immediately after each interview and were taken into account by researchers during the data analysis. Reflexive thinking was employed throughout the research process to reduce unintentional personal bias and enhance the trustworthiness of the study. The participants did not provide feedback regarding the findings. Data collection ceased only when data saturation was achieved. Therefore, the data collection and analysis processes were conducted simultaneously.

The interview guide was developed prior to conducting the interviews and was reviewed by a bioethicist (PV) and a psychologist and qualitative researcher (MA). To ensure consistency with the research question of this study, we developed the interview schedule by reference to the “integrated definition and dimensional schematic model” proposed by Mooney, Doyle and Ulrich with regard to the concept of parental moral distress. Mooney, Doyle and Ulrich noted that the overarching perspective on the concept of parent moral distress maintains that “parents experience relational solace and distress because of the impact of their child’s illness on relationships with themselves, their children, family, healthcare providers, their surrounding communities, and society” [5]. The interview guide was slightly refined after the initial results of a few interviews to improve its perspicacity.

The interview guide used in this study was developed for this study and included the following questions: (a) Please, would you like to describe in detail what it is like for you to be a parent of an infant hospitalized in the NICU? (*this item was a grand tour question intended to make the participant comfortable*). (b) Please, would you like to describe in detail the impact of their child’s illness on yourself and especially on your emotions? and the individuals surrounding them? (c) Please, would you like to describe in detail the impact of their child’s illness on your relationships with the individuals surrounding you i.e. with other members of your family, such as your partner/spouse, parents or other children (if any)? (d) During the hospitalization of your infant in the NICU, have you ever felt failed to behave in accordance with what you considered to be right? [If yes] Please describe your feelings and why did you feel so in detail. (e) During the hospitalization of your infant in the NICU, have you ever perceived that you were involved in situations in which you did not know what to do or decide? [If yes] Please, would you like to describe your emotions or experiences in response to these situations in detail? (f) During the hospitalization of your infant in the NICU, have you ever felt failed to engage in a medical decision-making process to the extent to which, and in a manner in which you would feel satisfied? [If yes] Please describe why did you feel so and your feelings in detail. (g) What was your relationship with NICU staff during the hospitalization of your infant? i) Do you have anything else to add?

Thematic analysis was used as the methodological framework of this study. Data analysis was conducted by the authors PV, MD and AKT, based on Mooney-Doyle and Ulrich’s integrated definition of parental moral distress. In the present study, we did not aim to conduct a conceptual analysis of moral distress. Therefore, we used an deductive approach to thematic analysis. We relied on preconceived themes by applying a theoretical framework based on existing knowledge. (Mooney-Doyle and Ulrich’s definition of parent moral distress). The interviews were audio-recorded and then transcribed verbatim. After carefully reading and rereading each interview transcript, the researchers coded units that were similar in meaning. Codes featuring similar meanings were grouped into subcategories. Then, these subcategories were condensed into categories, which in turn were grouped into themes. To enhance the analysis further, the entire coding process was aided and organized using computer-assisted qualitative data analysis software (CAQDAS) (NVIVO, 2015). Disagreements among the authors were addressed through discussion.

### Techniques used to ensure rigor and trustworthiness

To ensure rigor and trustworthiness in this study, we relied on the methodology described by Lincoln and Guba, which requires the consideration of four key criteria: credibility, transferability, dependability and confirmability [26]. To enhance both the credibility and the confirmability of this study, we used a) the technique of ‘peer debriefing’. The first author (PV) and the psychologist author (MA) served as peer debriefers when examining the findings. B) We also used the technique of ‘investigator triangulation’. To address possible interpretation biases, a discussion was conducted. The field notes from the interviews were recorded, and reflexive dialog was included in the data analysis to ensure trustworthiness (credibility) and confirmability. The progress of the research was monitored weekly by the research team.

## Results

The participants’ ages ranged from 25 to 41 years. All participants were married or in a civil partnership. Nine of the fifteen participants had no other children. Three participants had two other children, and two other participants had one other child. Interestingly, the sample included one participant who had two infants in the (same) NICU at the time of the interview.

The participants’ characteristics are presented in Table 1.

**Table 1:** Participant characteristics

Participant	Age	Gender	Other Children
P1	34	Female	No
P2	38	Female	Yes (2)
P3	26	Female	No
P4	28	Female	Two (2) infants (twins) hospitalized in the NICU at the time of interview.
P5	32	Female	No
P6	31	Male	No
P7	37	Female	No
P8	25	Female	No
P9	36	Female	Yes (2)
P10	36	Female	No
P11	29	Female	No
P12	32	Female	No
P13	29	Female	Yes (2)
P14	31	Female	Yes (1)
P15	41	Female	Yes (1)

The thematic data analysis revealed three major themes and seven subthemes (Table 2).

**Table 2:** Major themes and subthemes

Theme	Subtheme
1. **While parents strongly desire to fulfill their roles, their desire energy cannot be converted to action (“dynamic” intrapersonal dimension).**	1.1. **Self-directed negative feelings were experienced by parents due to their inability to fulfill their caregiving/parental roles.** “…made me feel guilty about neglecting my other children and family members.” 1.2. **Intense internal conflict was experienced by parents in response to a moral dilemma that was difficult, which was perceived as irresolvable.** “There were times when I had to make difficult decisions, weighing the options and constantly questioning myself. The fear of making the wrong choice gnawed at me. It was a heavy load to carry, full of doubts, tears, and sleepless nights…”
2. **Parents experience self-directed negative feelings which are not related to desire energy (“static” intrapersonal dimension).**	2.1. **Objectively unjustified, self-directed negative feelings of guilt or failure were experienced by parents.** “Yes, because I had diabetes, I felt guilty. My husband keeps saying it’s not my fault, but I believe he feels differently on the inside. It might be my fault…” [bursts into tears]. 2.2. **Parents experienced moral distress due to the poor image of the ill infants.** “…they had to intubate him, and I didn’t want to see him that way… I asked them ‘Isn’t a simple incubator is not enough?’. They told me that it was necessary…”
3. **Parental moral distress features an interpersonal dimension.**	3.1. **Inadequate information may predispose parents to experience moral distress.***“…I wondered if the medical decisions made for my baby were the right ones. It was hard to fully trust the doctors and nurses when I felt like I didn’t fully understand what was going on”.* 3.2. **Neonatologists’ caring behaviors were unduly perceived by parents as paternalistic behaviors.** *“While I had a good relationship with the doctors, at one point, my opinion was not heard…I felt like something was crushing me…like my hands were tied…. I felt like an outsider in my own child’s medical journey.”* *“…sometimes I felt like [the doctors] were making decisions without taking my input into account; I can’t tell you exactly, but then I felt calm afterward knowing that they are doing the best for my child.”* 3.3. **Reasonable or justified institutional rules were unduly perceived by parents as constraints** *“…[I was hindered by] medical protocols…mechanisms surrounding my child… It broke my heart and brought tears to my eyes…”*

### While parents strongly desire to fulfill their roles, their desire energy cannot be converted to action (“dynamic” intrapersonal dimension)

While participants wanted to act or make a choice for the benefit of someone else, they were prevented from doing so because of constraints or uncertainty, which caused them to experience self-directed negative feelings.

#### Self-directed negative feelings experienced by parents due to their inability to fulfill their caregiving/parental roles

The data analysis revealed that participants experienced intense internal conflict due to their perceived inability to fulfill their parental roles successfully. This finding was recurring. Participant P15 experienced a dramatic internal conflict, which was perceived as insolvable (inescapable). As she noted,

The constant need to be in the NICU and my attachment to my hospitalized child sometimes made me feel guilty about neglecting my other children and family members. The guilt weighed heavily on me, and I often found myself overcome with emotion, stopping to wipe away tears as I faced the feeling of not being able to give my all to everyone who depended on me. [Additionally, regarding the hospitalized child] I was blaming myself for the situation…thinking about what I could have done differently to prevent this situation. The burden of responsibility was heavy, and there were times when I felt like I was failing as a parent. Participant 14 raised a similar point.

Furthermore, Participant P7 experienced intense internal conflict, which, however, was not perceived as insolvable. As she noted,

There were times when I had to decide whether to stay with my baby or go home to spend time with my other son; he is 8 years old… he definitely needs me. It was a hard decision to make, and I often felt guilty no matter what I chose…It’s hard to be there for everyone all the time, but I remind myself that we’re all doing our best’ (P7).

Moreover, Participant P11 experienced internal conflict, which, however, was not perceived as insolvable. The data analysis (of the interviews and field notes) gave us the impression that the conflict experienced by the participants was not intense. As she noted,

I wrestled with the decision of whether to hire a nanny to help take care of my baby once he was home. I wanted to be there for my child, but I also had to take care of myself and my own needs…I definitely felt like I wasn’t doing enough for everyone, but I know our baby in the NICU needs us more right now.”

Interestingly, while Participants P2, P5, P7, P11, and P13 expressed negative feelings resulting from their deep concern about their other children, they indicated that they had already developed coping mechanisms to alleviate this distress. As Participant P13 said, *“I remind myself that we are all in this together and we will get through this as a family.”* Participant P7 mentioned that “I keep reminding myself that we are all doing the best we can. Participants P2, P5 and P11 clearly declared that they felt that their ill children were the ones who urgent needed their parents at that time. More precisely, Participant P2 set priorities, noting that.

First, it’s this baby, then my other children, then my husband, and then me [pause] at the end. I love them so much and let it be me in the end, I don’t mind [smiles].

Finally, participants felt negative emotions of guilt due to the fact that the presence of an infant in the NICU prevented them from supporting their parents or partner (P1) or even from caring for themselves (P8).

#### Intense internal conflict was experienced by parents in response to a moral dilemma that was difficult, which was perceived as irresolvable

Participants declared that they experienced intense internal conflict due to the dilemmas they faced and perceived as insolvable (inescapable) in their current circumstances (mostly against the backdrop of extreme uncertainty). They felt that they were embedded in these dilemmas. The following quotation from the interview with Participant 15 is representative of that point. This participant experienced very intense internal conflict. As she noted,

There were times when I had to make difficult decisions, weighing the options and constantly questioning myself. The fear of making the wrong choice gnawed at me. It was a heavy load to carry, full of doubts, tears, and sleepless nights…the conflicting information and emotions made it incredibly difficult.

Participant 14 made similar comments.

Participant P3 had difficulty weighing her options to make a decision regarding the care of her baby. As she noted,

I was struggling with the decision of whether or not to breastfeed him. While I knew it was the best thing for my baby, I had to weigh that against the physical and emotional toll it took. It’s so new, and I don’t know how to handle it.

### Parents experience self-directed negative feelings which are not related to desire energy (“static” intrapersonal dimension)

Participants experienced self-directed negative feelings in response to morally undesirable situations without, however, wanting to act (or needing to make a choice) for the benefit of someone else.

#### Objectively unjustified, self-directed negative feelings of guilt or failure were experienced by parents

The participants felt guilty about their inability to protect their infants from illness or suffering, despite the fact that their feelings of guilt were not based on logical reasons or clear thinking. This finding was recurring. Participants P1, P2, P3, and P11 indicated that they experienced negative self-directed emotions of guilt and self-criticism due to their unhealthy state and lifestyle. As Participant P1 noted,

I thought that something was wrong with my body. I thought it was my body’s fault for my baby’s condition and that is was my fault that it’s in there now.

Similarly, Participant P2 provided the following confession:

Because I had gestational diabetes, I was told not to eat some sweets, and I didn’t pay enough attention, I don’t know, maybe I’m to blame…if I didn’t eat them, I don’t know….

Similarly, as Participant P3 declared,

Yes, because I had diabetes, I felt guilty. My husband keeps saying it’s not my fault, but I believe he feels differently on the inside. It might be my fault…[bursts into tears].

Likewise, Participant P11 made the following comment:

I blamed myself for not noticing the signs of early labor and being too late to come here.

Participant P6 experienced negative self-directed emotions of anger because he perceived that he had outlined the birth plan incorrectly. As he noted,

I was angry with myself for not being able to provide the best medical care for my infant because we came from Kozani [a town in northern Greece] by ambulance. Of course, I knew she would give birth here, but I didn’t plan it properly.

Participants P6, P8, P10, P12, P13 and P14 experienced negative self-directed emotions of guilt, anger, self-criticism, and low self-esteem due to their inability to do otherwise, despite the fact that they were left with no choice and were aware of this fact. As they noted,

I know that it’s not my fault, but the role of a parent awakened in me. I felt I didn’t protect my infant… (P8). I was angry with myself for not being able to give the baby a healthy start in life, but I know that it’s not my fault (P10). I felt guilty that I couldn’t breastfeed my baby, even though I knew that I shouldn’t (P12). There were times when I felt like I was failing as a parent because I couldn’t be there for my baby 24 h a day (P13). When my baby had to be intubated, I was torn between feeling grateful that he was getting the medical care he needed and feeling guilty that I couldn’t do more to protect him (P5). Guilt was a frequent companion during our journey to the ICU. I blamed myself for the circumstances that led to my child’s hospitalization, constantly asking if there was anything I could have done differently. The guilt intensified when I saw my child’s pain or setbacks, and I often blamed myself for not being able to protect them from these struggles (P14).

Participant P11 dramatically declared her feelings of failure as follows:

You know what, Maria, it was really frustrating because I just wanted to do the best for my child [pause], but I couldn’t [bursts into tears].

#### Parents experienced moral distress due to the poor image of the ill infants

Participants P2, P3 and P5 felt constrained by themselves. They wanted to act on what they knew to be right. However, they feared that acting in this manner might harm themselves.

As Participant P2 noted,

There were times when I didn’t know which was right. For example, when my baby needed a medical procedure, they had to intubate him, and I didn’t want to see him that way. I asked them “Isn’t a simple incubator is not enough?“. They told me that it was necessary….

As Participant P5 noted,

Yes, there were definitely times when I felt like something was holding me back from taking the actions or decisions that I knew were best for my child. Sometimes when I went to see it, I was afraid that I would be frightened by the image or that it would be forever etched in my mind.

### Parental moral distress features an interpersonal dimension

#### Inadequate information may predispose parents to experience moral distress

Well-informed parents developed better relationships with physicians (thus protecting parents from experiencing moral distress). Participants claimed to be involved in medical decisions, but they noted that they needed to be sufficiently well informed to do so. A lack of information may be an external constraint that can serve as an antecedent of parental moral distress. The following quotation is representative of this point. Participant P3 mentioned that if she were adequately informed, she could be involved in medical decisions more effectively:,

There were definitely times when I felt like my hands were tied when it came to making decisions about my child in the NICU. It was really frustrating because I wanted to be more involved in the process, but I just didn’t feel like I had enough information to do so.

Furthermore, Participant P9 noted that the lack of information provided to her undermined her trust in health care professionals. As she noted,

At times, I wondered if the medical decisions made for my baby were the right ones. It was hard to fully trust the doctors and nurses when I felt like I didn’t fully understand what was going on.

### Neonatologists’ caring behaviors were unduly perceived by parents as paternalistic behaviors

This subtheme was mainly about the participants’ unreasonable or unjustified with to be involved in decision making process in dilemmas that were outside of the so-called zone-of-parental discretion. What parents perceived as paternalistic behavior was not actually paternalistic for two reasons presented below.

First. Participants clearly indicated that medical paternalism and institutional factors, including institutional rules, could serve as constraints that could prevent parents from making decisions regarding their infant’s care or taking action based on what they knew to be right. It should be noted however that while participants felt they were partially or wholly excluded from the decision-making process, they did not mention specific situations where their views about treatment for their infants were strongly opposed to physicians’ views.

Participant P14 focused on medical paternalism. As she noted,

My relationship with the doctors and staff was positive. [However,] there was one particular instance where I disagreed with a treatment plan, believing that it could potentially do more harm than good. However, my concerns were dismissed, and I was left feeling weak and disappointed.

Similarly, Participant P15 clearly emphasized her experience of medical paternalism. As she noted,

While I had a good relationship with the doctors, at one point, my opinion was not heard…I felt like something was crushing me…like my hands were tied…. I felt like an outsider in my own child’s medical journey.

Furthermore, Participants P6, P7, P9, P10 and P12 indicated that while they desired greater parental involvement in decision-making, they could not make decisions regarding their infants’ care or take action based on what they believed to be right. They mentioned that they were partially or wholly excluded from the decision-making process. In other words, they highlighted their experiences of medical paternalism. Participant P6 claimed that such constraints may arise from a range of factors, including medical paternalism and hospital policy or administrative procedures. Finally, Participant P9 reported that constraints may arise from physicians (through medical paternalism or the provision of inadequate information).

Second. Parents initially expressed mild concerns regarding the physicians’ choices, which, however, were eventually eliminated.

Most participants reported that they had excellent relationships with the NICU physicians and trusted them. However, some participants noted that they had initially raised mild and minor concerns regarding their infant’s care, which then faded away, either because parents realized that neonatologists knew better and had done the right thing or because parents wondered whether their concerns were well-grounded and would promote the infant’s best interest. Perhaps parents experienced some degree of mild moral distress (due to the perceived external constraint of “my voice is not heard”), which then, however, faded away.

As Participant P2 said,

…sometimes I felt like [the doctors] were making decisions without taking my input into account; I can’t tell you exactly, but then I felt calm afterward knowing that they are doing the best for my child.

Similarly, Participant P3 made the following statement:

My relationship with the doctors and staff was generally good, but there was one instance where I felt that my opinion was not taken into account. I wanted my child to be discharged from the hospital, but the doctors felt it was too soon.…I understand they were trying to do the best for my baby, but it was frustrating not to have my opinion heard. I’m not sure. Maybe I wasn’t thinking straight.

Similarly, as Participant P6 said,

I have a good relationship with the doctors and staff, but there was a situation where I felt like I wasn’t being listened to. I wanted my child to receive a certain type of milk… I don’t know if it was the right one, but anyway.”

Importantly, parents must observe physicians working with devotion and doing their best. The need to observe physicians in this way was identified through the data analysis as a factor that could enhance trust between parents and neonatologists.

As Participant P4 said,

This is not just for show; I know that they are doing the right thing, and I trust them.

Subsequently, the same participant added the following:

I preferred live communication. I wanted to be here to see them and hear them and understand them. The doctors supported me… I trust them.

Furthermore, as Participant P2 said,

They were amazing during this difficult time. There was never a case where I felt that my wishes were not respected. Everyone was just running around and I couldn’t keep track of what was going on….

### Reasonable or justified institutional rules were unduly perceived by parents as constraints

Participant P14 focused on institutional rules (protocols):

There were times in the ICU when I felt an overwhelming sense of helplessness. … I wanted to hold my baby in my arms, comfort him and offer him that human touch…what my instincts urged me to do…I knew this would bring him comfort, but the medical staff advised against it due to his fragile condition …[I was hindered by] medical protocols…mechanisms surrounding my child… It broke my heart and brought tears to my eyes….

Similarly, Participant P8 focused on hospital visitation restrictions in Greek hospitals during the COVID-19 pandemic.

Furthermore, Participant P6 noted that constraints may arise from *hospital policy or administrative procedures.*

## Discussion

According to Mooney-Doyle and Ulrich’s integrated definition of parental moral distress, such distress involves “an intrapersonal dimension, an interpersonal dimension, and a spiritual/existential dimension” [5]. Consistent with Mooney-Doyle and Ulrich’s approach to parental distress, we identified intrapersonal and interpersonal dimensions of parental moral distress.

However, in the present study, a distinction between the dynamic intrapersonal dimension and the static intrapersonal dimension of parental moral distress emerged from the data analysis. With respect to what we call the “dynamic” intrapersonal dimension, while parents want to act (or must make a choice) for the benefit of someone else, they are prevented from doing so because of constraints or uncertainty, which causes them to experience self-directed negative feelings. With respect to what we call the “static” intrapersonal dimension, parents experience self-directed negative feelings in response to morally undesirable situations without, however, wanting to act (or needing to make a choice) for the benefit of someone else. In other words, with respect to what we call the “dynamic” intrapersonal dimension of parent moral distress, parents constantly experience self-directed negative feelings caused by the fact that external constraints or inner struggle (in case of high uncertainty) frustrate their strong desire to care (or participate in caring) about someone else (i.e. the ill baby or a family member). With respect to what we call the “static” intrapersonal dimension of parent moral distress, parents experience self-directed negative feelings which are reaction to a stationary situation that does not involve parent’s struggle to take an active or more active role in caring about someone else.

The “dynamic” intrapersonal dimension refers to the inhibition of an action that is consistently desired by parents. The impact of a hospitalized infant’s illness on other children in the same family can cause parents to experience “indirect moral distress” because they feel that they are unable to meet the basic needs of all their children successfully. They feel that they are not in a position to fulfill their perceived parental duties successfully because “no one is getting what they need and won’t be for a while” [5, 27]. Furthermore, it should be noted that parents perceive the meaning of being a “good parent” in the NICU environment differently [28, 29]. Parents spend considerable amounts of energy and time with their hospitalized baby. They are not capable of being in more than one place simultaneously. Therefore, they experience “inner conflict and turmoil” [5]. As certain primary moral similarities are evident between this feeling of “inner conflict and turmoil” and moral constraint distress, the feeling of “inner conflict and turmoil” can be labeled moral distress [5, 30]. Both experiences entail morally troubling situations that can “similarly violate core moral values”. Note that this expression has been used in the literature to suggest moral similarities between moral constraint distress and moral uncertainty distress [12]. In that regard, it should be noted that parents’ involvement in medical decisions regarding their infant’s care can strengthen their perceived ability to fulfill their parental roles [7, 31, 32]. Furthermore, parents can experience “inner conflict and turmoil” when they are embedded in a morally troubling situation in which they must resolve a difficult ethical dilemma. While they strive to act for the benefit of someone else, they are prevented from doing so due to extremely high uncertainty.

Two participants in this study reported that they experienced intense conflict in their inner worlds because they were focused on difficult ethical dilemmas that they perceived as insolvable (inescapable). The participants faced a difficult moral dilemma that they perceived to be irresolvable (although this was not truly the case), and they seemed to experience intense internal conflict (“inner conflict and turmoil”) [5]. Similar situations were reported in a recent study conducted in Greece [33]. Intense internal conflict entails high levels of what Mooney-Doyle and Ulrich referred to as “direct moral distress”. According to these authors, parents may feel compelled to make “agonizing” decisions for their infant hospitalized in the NICU (especially in cases featuring extremely premature infants). This situation is what those authors called direct moral distress [5].

The static intrapersonal dimension of parental moral distress focuses exclusively on parents’ inner worlds. Parents do not strive to act for the benefit of someone else in this context.

Mothers of preterm neonates may feel sadness and guilt due to the fact that they delivered their infants prematurely [34]. Mothers of preterm infants who are hospitalized in the NICU experience significantly higher levels of postpartum depression and guilt [35]. Furthermore, it has been argued that after the death of a child in the neonatal period, “parents often experience feelings of guilt, disenfranchisement, feelings of betrayal by one’s own body” [36]. These negative emotions are objectively unjustified given that in reality, mothers bear no responsibility for the aforementioned health conditions of their infants.

The interpersonal dimension of parental moral distress implies that a negative relationship between parents and health care professionals can cause parents to experience moral distress, whereas positive relationships between parents and health care professionals can protect parents from moral distress [5]. Mooney-Doyle and Ulrich claimed that the quality of relationships between parents and health care providers can affect parents deeply because these relationships are “vital to their child’s health, their own emotional health, their family health, and their ability to be the parent they want to be to their seriously ill child” [5]. Suboptimal/ineffective communication between parents and health care professionals can serve as a significant antecedent of moral distress, and compassionate physicians who make every honest effort to care for ill infants and support (and inform) parents can protect against such parental moral distress [5].

In the present study, most participants perceived health care professionals positively. The majority of participants did not report poor relationships with NICU physicians. None of the participants in the present study reported any substantial disagreements between parents and physicians. In that regard, it has been argued that perceived disagreements between NICU physicians and parents are very rare and mostly occur in cases of parents who “want too much” [2]. Furthermore, the parents’ perceptions of physicians who went beyond the call of duty and made every honest effort to ensure optimal outcomes for the ill infant were identified as a trust-building factor. This finding is consistent with the findings of a similar qualitative study conducted recently in Greece [33]. Note, however, that in that previous study, most participants identified physician fatigue as a barrier to physicians’ ability to exert the utmost effort to assist the hospitalized infant [33].

The participants in this study emphasized their lived experiences of paternalistic care behavior. This finding is partly consistent with the findings of a similar study previously conducted in Greece, although in that study, most participants emphasized the fact that the physicians were caring for their baby on the basis of the belief that they knew what is best for the infant [33]. In the present study, participants complained that they were not offered the opportunity to provide input regarding the care of their ill infant. They felt that their voices were not heard by the physicians. However, this feeling was not a reaction to a specific situation involving actual paternalism. It was rather based on the participants’ general belief that they should play a more active role in promoting the best interest of the infant (P2, P5, P7, P9, P15). Participant P14’s desire to provide such input, which was not honored, was based on intuition. Furthermore, participants reported constraints, which, however, pertained to matters that were clearly within the zone of physician discretion and were therefore not reasonable or justified constraints. Participant P14 reported barriers to hugging and touching the infant(which, in her view, would have been doing the right thing). Participant P3 disagreed with the physicians’ decision to keep the baby in the hospital, and another participant (P6) disagreed with the physicians regarding the type of milk to be provided to the infant. The behaviors identified as paternalistic pertained to issues that did not fall within the zone of parental discretion. At any rate, it should be noted that all participants either explicitly stated or implied that they had good interactions with the physicians and trusted them.

Interestingly, while some participants felt that they were powerless to act according to what they believed to be the right thing because of (perceived) mild medical paternalism, they did not feel isolated or perceive that their infant was undervalued. Note, however, as Mooney-Doyle and Ulrich have previously indicated, that “Parents may perceive they are isolated, feel powerless to make a decision about their infant’s care and fear their infant is undervalued” [5].

Regarding paternalism, most participants in this study expressed their desire to be further involved in medical decisions regarding the infant’s care. This parental attitude has been strongly emphasized in the literature. It has been argued that most parents perceive their involvement in decision-making as part of their parental role, especially with respect to high-risk decisions or “decisions perceived to be part of the normal parental role” [37, 38]. However, preferences concerning involvement in decision-making vary widely among parents with an infant in the NICU [39, 40]. Furthermore, the fear of decisional burden may affect parents in different ways [40]. Importantly, parents’ involvement in decision-making has not yet been fully explored [41]. To ensure that parents are meaningfully involved and that their views are equally represented and reflected in the context of shared decision-making, neonatologists should seek feedback from parents regarding how they want to be involved. In that regard, it has been argued that the ethics associated with the challenging situation of disagreement between parents and NICU treatment teams concerning appropriate neonatal treatment require the application of an ethical tool known as the “zone of parental discretion”, which calls for a balance between infants’ well-being and parents’ right to make decisions for their infant [42]. As previous research has shown that it is difficult to achieve such a balance in practice, it is suggested that an ethics framework would be helpful in this context [2]. More precisely, it has been argued that parents can choose between life-sustaining treatment and palliative care in cases featuring infants who are on the borderline of viability, irrespective of the neonatologists’ own personal values. This so-called “gray zone” is defined in terms of prognosis (on the basis of a range of prognostic factors) rather than in terms of gestational age [43].

At any rate, parents want to be provided with adequate, balanced and individualized information to feel that they are able to be sufficiently involved in decisions concerning their infant’s care [44]. This finding also emerged in this study. In this study, parents initially expressed mild concerns that subsequently faded away. They felt that they had incomplete or missing information or were uncertain about their knowledge, including with regard to the quality of life of their infants. Indeed, it is difficult for ill-informed parents to make decisions regarding difficult dilemmas involving the quality of life of their ultimately surviving infants. It is true that “parents are generally not informed about the evidence regarding the quality of life of surviving infants” [45]. Note, however, that the quality of life of a premature infant who is hospitalized in the NICU can hardly be understood without vagueness [46]. Furthermore, Saigal et al. concluded that “at young adulthood, health-related quality of life was not related to size at birth or to the presence of disability” [47]. However, perceived quality of life is a significant determinant affecting parents’ decisions, which are difficult in cases featuring extreme uncertainty, such as in the neonatal intensive care context [8, 48]. Neonatal intensive care is a context involving a high degree of uncertainty. Parental uncertainty is an experience that is ‘uniquely different’ from other experiences [49].

The degree of medical paternalism observed in the NICU varies across countries. For instance, in the United States, the model of parental autonomy prevails, whereas in France, the model of medical paternalism prevails [50]. It has been argued that medical paternalism prevails in end-of-life decisions in Greece [51]. The intuitions of Greek physicians who are committed to the traditional values of Hippocratic professional ethics are oriented toward the role of the healer [52]. Medical paternalism in the NICU context was not observed in this study.

Furthermore, preventing mothers from providing their infants with their own breast milk or engaging in skin-to-skin contact with them were identified as sources of self-directed negative emotions that compromised the parents’ moral integrity or values, namely, as sources of parental moral distress. A similar finding emerged from a previous study conducted recently in Greece [33]. It should be noted, however, that in the present study, these emotions were not reasonable or justifiable. These constraints were appropriate for critically ill infants hospitalized in the NICU, especially at a time when they faced the risk of transmission of the coronavirus. It is true that “mothers who are unable to meet their breastfeeding goals are at higher risk for anxiety, depression, embarrassment, and guilt” [53]. In cases featuring a high risk of the mother-to-child transmission of an infectious disease, mothers reported experiencing sadness and guilt for their failure to breastfeed, while they regarded breastfeeding as a way of developing bonds with their infants and benefitting them [54]. Furthermore, it has been argued that mothers’ feelings of guilt and shame are associated with formula postpartum infant feeding practices [55]. As mentioned, the literature has highlighted the fact that mothers of infants in the NICU experience a sense of failure or guilt because they delivered the infants prematurely [33, 56]. Mothers’ feelings of connection with their infants (i.e., through breastfeeding or engaging in skin-to-skin contact with them) can ease mothers’ sense of failure or guilt [56, 57]. Preventing mothers from providing milk for their infants or engaging in skin-to-skin contact with them are inherent stressors in the neonatal intensive care context [56, 58, 59]. It has been argued that mothers prioritize infants’ needs over their own needs and strive to provide their infants who are hospitalized in the NICU with their own breast milk despite multiple possible barriers [6]. Furthermore, it has been argued that parents’ unrestricted physical proximity to their neonates (24 h per day) enables them to feel respected and helps them ‘achieve independent parenthood at the time of discharge’ [57].

Moreover, the present study did not reveal that parental values related to religiosity/spirituality cause disagreement between parents and physicians. Conversely, a study conducted previously in Greece reported that deeply held spiritual and/or existential values on the part of parents can result in strong disagreements between parents and physicians [33]. Almost half of the participants in this research emphasized the fact that their decisions or relationships with physicians were not affected by factors related to religion or spirituality (P2, P3, P6, P7, P9, P10, P12). Interestingly, almost half of the participants in this study indicated that their religiosity/spirituality and their trust in God had made them stronger or more competent to make decisions and trust physicians (P1, P4, P5, P8, P11, P13, P14, P15).

Finally, a study previously conducted in Greece reported that the fear of taking responsibility for decisions deters parents from becoming involved in the shared decision-making process [33]. This finding was in line with prior studies [38]. However, this finding did not emerge in the present study.

The present study has implications for practice and research. The findings of this study provide further insights into the complex and still ill-defined phenomenon of parent moral distress as defined by Mooney-Doyle and Ulrich. The study introduces new information, which might serve as starting point for future research. Furthermore, the findings of the present study contribute to a more comprehensive understanding of how an infant’s serious illness impacts on parents. The study points out these impacts. Therefore, these findings might contribute to the development of interventions to promote family-centered care in the NICU context. In the dehumanizing hi-tech NICU environment the successful implementation of family-centered care is challenging. Nevertheless, it should be further endorsed by health care organizations and policymakers.

## Limitations

This study should be interpreted in light of certain limitations. The study was conducted through consultation with parents with infants who were hospitalized in only one NICU. Note, however, that in this NICU, infants were admitted from various regions of northern Greece. Furthermore, another limitation of this study is that only one of the fifteen participants was male (the father). Moreover, social desirability bias or authority bias cannot be excluded. Physicians may exhibit an authoritarian attitude, thus indicating a power imbalance in the physician-parent relationship. Finally, participants were not asked to check the consistency between their intentions and the results obtained by the researchers. This situation limits the reliability of the study in terms of confirmability.

## Conclusion

In general, the results of this study support the integrated definition of parental moral distress proposed by Mooney-Doyle and Ulrich. Furthermore, the present study introduces new information. The study distinguishes between the dynamic and static aspects of the intrapersonal dimension of the phenomenon of parental moral distress. With respect to what we call the “dynamic” intrapersonal dimension, while parents strongly desire to fulfill their roles, their desire energy cannot be converted to action). In the “static” intrapersonal dimension parents experience self-directed negative feelings, which however, are not related to desire energy. Moreover, participants experienced moral distress because they unduly perceived certain situations as causing moral distress. In addition, inadequate information may predispose parents to experience moral distress. The findings of the present study may contribute promote family-centered care in the NICU context. These findings may contribute to the creation of a more responsive health care system featuring sensitive support systems that can help parents make morally sound judgments for their infants in the traumatic NICU context.

## Data Availability

The transcripts of the full interviews that were collected and qualitatively analysed in the current study are not available for reasons of confidentiality. The redacted transcripts that were used and analysed as part of the current study can be made available by the corresponding author upon reasonable request.

## Abbreviations

NICU: Neonatal Intensive Care Unit
EPI: Extremely Premature Infant

## References

[1] Janvier A, Lantos J, Aschner J, Barrington K, Batton B, Batton D et al. Stronger and More Vulnerable: A Balanced View of the Impacts of the NICU Experience on Parents. Pediatrics. 2016;138(3):e20160655. 10.1542/peds.2016-0655. Epub 2016 Aug 3. PMID: 27489297. 10.1542/peds.2016-0655 27489297

[2] Prentice TM, Janvier A, Gillam L, Donath S, Davis PG. Moral Distress in Neonatology. Pediatrics. 2021;148(2): e2020031864. doi: 10.1542/peds.2020–031864. Epub 2021 Jul 20. PMID: 34285081. 10.1542/peds.2020-031864 34285081

[3] Staver MA, Moore TA, Hanna KM (2021). An integrative review of maternal distress during neonatal intensive care hospitalization. Arch Womens Ment Health.

[4] Staver MA, Moore TA, Hanna KM. Maternal Distress in the Neonatal Intensive Care Unit: A Concept Analysis. Adv Neonatal Care 2019;19(5):394–401. 10.1097/ANC.0000000000000642. PMID: 31306234. 10.1097/ANC.0000000000000642 31306234

[5] Mooney-Doyle K, Ulrich CM. Parent moral distress in serious pediatric illness: A dimensional analysis. Nurs Ethics 2020;27(3):821–837. doi: 10.1177/0969733019878838. Epub 2020 Mar 5. PMID: 32138577. 10.1177/0969733019878838 32138577

[6] Rossman B, Greene MM, Kratovil AL, Meier PP. Resilience in mothers of very-low-birth-weight infants hospitalized in the NICU. J Obstet Gynecol Neonatal Nurs. 2017 May-Jun;46(3):434–45. Epub 2017 Mar 2. PMID: 28263724. 10.1016/j.jogn.2016.11.016 28263724

[7] Loewenstein K. Parent psychological distress in the neonatal Intensive Care Unit within the context of the Social Ecological Model: a scoping review. J Am Psychiatr Nurses Assoc. 2018 Nov/Dec;24(6):495–509. 10.1177/1078390318765205. Epub 2018 Mar 26. PMID: 29577790. 10.1177/1078390318765205 29577790

[8] Mills M, Cortezzo DE (2020). Moral Distress in the neonatal intensive care unit: what is it, why it happens, and how we can address it. Front Pediatr.

[9] Kvalnes Ø. Moral Reasoning at Work Rethinking Ethics in Organizations. Second Edition. Palgrave Pivot; 2nd ed 2019 edition (April 26, 2019), p. 11.

[10] Bolívar Montes LÁ, Montalvo Prieto A. Uncertainty Associated to Parents of Preterm Infants Hospitalized in Neonatal Intensive Care Units. Invest Educ Enferm. 2016;34(2):360–367. 10.1590/S0120-072016000200016. PMID: 28569940. 10.1590/S0120-53072016000200016 28569940

[11] Parish O, Williams D, Odd D, Joseph-Williams N. Barriers and facilitators to shared decision-making in neonatal medicine: A systematic review and thematic synthesis of parental perceptions. Patient Educ Couns 2021 Aug 27:S0738-3991(21)00579-6. 10.1016/j. pec.2021.08.033. Epub ahead of print. PMID: 34503868. 10.1016/j.pec.2021.08.033 34503868

[12] Fourie C. Who Is Experiencing What Kind of Moral Distress? Distinctions for Moving from a Narrow to a Broad Definition of Moral Distress. AMA J Ethics. 2017;19(6):578–584. 10.1001/journalofethics.2017.19.6.nlit1-1706. PMID: 28644787. 10.1001/journalofethics.2017.19.6.nlit1-1706 28644787

[13] Ramathuba DU, Ndou H (2020). Ethical conflicts experienced by intensive care unit health professionals in a regional hospital, Limpopo province, South Africa. Health SA.

[14] Wurzbach M-E. Experiencing Moral Uncertainty in Practice, Published online 11AD, 2015, http://hdl.handle.net/10755/581749 (last access: 8 June 2023).

[15] Kälvemark S, Höglund AT, Hansson MG, Westerholm P, Arnetz B. Living with conflicts-ethical dilemmas and moral distress in the health care system. Soc Sci Med 2004;58(6):1075-84. 10.1016/s0277-9536(03)00279-x. PMID: 14723903. 10.1016/s0277-9536(03)00279-x 14723903

[16] Jameton A (1984). Nursing practice: the ethical issues.

[17] Jameton A, Friedman E (1992). Nursing ethics and the moral situation of the nurse. Choices and conflict.

[18] Wilson CA, Metwally H, Heavner S, Kennedy AB, Britt TW (2022). Chronicling moral distress among healthcare providers during the COVID-19 pandemic: a longitudinal analysis of mental health strain, burnout, and maladaptive coping behaviours. Int J Ment Health Nurs.

[19] Prentice TM, Gillam L, Davis PG, Janvier A (2018). The use and misuse of moral distress in neonatology. Semin Fetal Neonatal Med.

[20] Morley G, Ives J, Bradbury-Jones C, Irvine F (2019). What is ‘moral distress’? A narrative synthesis of the literature. Nurs Ethics.

[21] Deschenes S, Gagnon M, Park T, Kunyk D (2020). Moral distress: a concept clarification. Nurs Ethics.

[22] Fourie C (2015). Moral distress and moral conflict in clinical ethics. Bioethics.

[23] Woods M (2014). Beyond moral distress: preserving the ethical integrity of nurses. Nurs Ethics.

[24] Foe G, Hellmann J, Greenberg RA (2018). Parental Moral Distress and Moral Schism in the neonatal ICU. J Bioeth Inq.

[25] Campbell SM, Ulrich CM, Grady C (2006). A broader understanding of moral distress. Am J Bioeth.

[26] Lincoln YS, Guba EG (1985). Naturalistic Inquiry.

[27] Mooney-Doyle K, Deatrick JA, Ulrich CM (2018). Parenting in childhood life-threatening illness: a mixed-methods study. J Palliat Med.

[28] October TW, Fisher KR, Feudtner C (2014). The parent perspective: being a good parent when making critical decisions in the PICU. Pediatr Crit Care Med.

[29] Feudtner C, Walter JK, Faerber JA (2015). Good-parent beliefs of parents of seriously ill children. JAMA Pediatr.

[30] Walsh JP (2018). Care, commitment and moral distress. Ethical Theory Moral Pract.

[31] Verberne LM, Kars MC, Schouten-van Meeteren AY (2017). Aims and tasks in parental caregiving for children receiving palliative care at home: a qualitative study. Eur J Pediatr.

[32] Aarthun A, Øymar KA, Akerjordet K (2018). Parental involvement in decision-making about their child’s health care at the hospital. Nurs Open.

[33] Voultsos P, Deligianni M, Tsamadou E. Parents’ experiences of uncertainty, (shared) decision-making and moral distress during their infants’ previous hospitalization in the NICU. Aristotle Biomedical J 2022, 4, 2 e-ISSN: 2653–9748.

[34] Rafael-Gutiérrez SS, García PE, Prellezo AS, Paulí LR, Del-Castillo BL, Sánchez RB. Emotional support for parents with premature children admitted to a neonatal intensive care unit: a qualitative phenomenological study. Turk J Pediatr. 2020;62(3):436–449. 10.24953/turkjped.2020.03.011. PMID: 32558418. 10.24953/turkjped.2020.03.011 32558418

[35] Park J, Bang KS (2022). The physical and emotional health of South Korean mothers of preterm infants in the early postpartum period: a descriptive correlational study. Child Health Nurs Res.

[36] Hvidtjørn D, Prinds C, Bliddal M, Henriksen TB, Cacciatore J, O’Connor M (2018). Life after the loss: protocol for a Danish longitudinal follow-up study unfolding life and grief after the death of a child during pregnancy from gestational week 14, during birth or in the first 4 weeks of life. BMJ Open.

[37] Gibbs G. The Sage qualitative research kit. Analyzing qualitative data. Sage Publications Ltd.; 2007.

[38] Weiss EM, Xie D, Cook N, Coughlin K, Joffe S (2018). Characteristics Associated with preferences for parent-centered decision making in neonatal intensive care. JAMA Pediatr.

[39] Pascale Blakey E, Jackson D, Walthall H, Aveyard H. Memory in narratives and stories: implications for nursing research. Nurse Res. 2019;27(3):27–32. 10.7748/nr.2019.e1641. PMID: 31524339. 10.7748/nr.2019.e1641 31524339

[40] Weiss EM, Magnus BE, Coughlin K (2019). Factors associated with decision-making preferences among parents of infants in neonatal intensive care. Acta Paediatr.

[41] Soltys F, Philpott-Streiff SE, Fuzzell L, Politi MC (2020). The importance of shared decision-making in the neonatal intensive care unit. J Perinatol.

[42] Gillam L (2016). The zone of parental discretion: an ethical tool for dealing with disagreement between parents and doctors about medical treatment for a child. Clin Ethics.

[43] Wilkinson D. Who should decide for critically ill neonates and how? The grey zone in neonatal treatment decisions. In: McDougall R, Delany C, Gillam L, editors. When Doctors and Parents Disagree: Ethics, Paediatrics & the Zone of Parental Discretion. Sydney (AU): The Federation Press; 2016 Jun 20. Chapter 4. PMID: 28661627. 28661627

[44] Staub K, Baardsnes J, Hebert N, Hebert M, Newell S, Pearce R (2014). Our child is not just a gestational age. A first-hand account of what parents want and need to know before premature birth. Acta Paediatr.

[45] Janvier A, Prentice T, Lantos J. Blowing the whistle: moral distress and advocacy for preterm infants and their families. Acta Paediatr 2017;106(6):853–854. 10.1111/apa.13852. PMID: 28489312. 10.1111/apa.13852 28489312

[46] Deligianni M, Voultsos P, Tzitiridou-Chatzopoulou MK, Drosou-Agakidou V, Tarlatzis V (2023). Moral distress among neonatologists working in neonatal intensive care units in Greece: a qualitative study. BMC Pediatr.

[47] Saigal S, Stoskopf B, Pinelli J, Streiner D, Hoult L, Paneth N, Goddeeris J. Self-perceived health-related quality of life of former extremely low birth weight infants at young adulthood. Pediatrics. 2006;118(3):1140-8. 10.1542/peds.2006-0119. PMID: 16951009. 10.1542/peds.2006-0119 16951009

[48] Sullivan A, Cummings C (2020). Historical perspectives: Shared decision making in the NICU. Neoreviews.

[49] Malin KJ, Johnson TS. A Concept Analysis of Parental Uncertainty in Illness of an Infant. MCN Am J Matern Child Nurs. 2019 Jul/Aug;44(4):206–211. 10.1097/NMC.0000000000000535. PMID: 31261298. 10.1097/NMC.0000000000000535 31261298

[50] Orfali K. Parental role in medical decision-making: fact or fiction? A comparative study of ethical dilemmas in French and American neonatal intensive care units. Soc Sci Med. 2004;58(10):2009-22. 10.1016/S0277-9536(03)00406-4. PMID: 15020016. 10.1016/S0277-9536(03)00406-4 15020016

[51] Kranidiotis G, Gerovasili V, Tasoulis A, Tripodaki E, Vasileiadis I, Magira E, Markaki V, Routsi C, Prekates A, Kyprianou T, Clouva-Molyvdas PM, Georgiadis G, Floros I, Karabinis A, Nanas S (2010). End-of-life decisions in Greek intensive care units: a multicenter cohort study. Crit Care.

[52] Sommerville A (2003). Juggling law, ethics, and intuition: practical answers to awkward questions. J Med Ethics.

[53] Elder M, Murphy L, Notestine S, Weber A (2022). Realigning expectations with reality: a case study on maternal Mental Health during a difficult breastfeeding journey. J Hum Lact.

[54] Carroll C, Booth A, Campbell F, Relton C (2020). Qualitative evidence synthesis of values and preferences to inform infant feeding in the context of non-HIV transmission risk. PLoS ONE.

[55] Jackson L, De Pascalis L, Harrold J, Fallon V (2021). Guilt, shame, and postpartum infant feeding outcomes: a systematic review. Matern Child Nutr.

[56] Rossman B, Kratovil AL, Greene MM, Engstrom JL, Meier PP. I have faith in my milk: the meaning of milk for mothers of very low birth weight infants hospitalized in the neonatal intensive care unit. J Hum Lact. 2013 Aug;29(3):359–65. Epub 2013 Apr 18. PMID: 23599267. 10.1177/0890334413484552 23599267

[57] Stelwagen M, van Kempen A, Westmaas A, Vet E, Scheele F. Parents’ Experiences With a Model of Integrated Maternity and Neonatal Care Designed to Empower Parents. J Obstet Gynecol Neonatal Nurs. 2021 Mar;50(2):181–92. Epub 2021 Jan 8. PMID: 33428875. 10.1016/j.jogn.2020.11.001 33428875

[58] Gonya J, Nelin LD (2013). Factors associated with maternal visitation and participation in skin-to-skin care in an all referral level IIIc NICU. Acta Paediatr.

[59] Greene MM, Rossman B, Patra K, Kratovil A, Khan S, Meier PP. Maternal psychological distress and visitation to the neonatal intensive care unit. Acta Paediatr 2015;104(7): e306-13. 10.1111/apa.12975. Epub 2015 Feb 27. PMID: 25684177; PMCID: PMC4792263. 10.1111/apa.12975 PMC4792263 25684177

